# Detecting longitudinal effects of haplotypes and smoking on hypertension using B-splines and Bayesian LASSO

**DOI:** 10.1186/1753-6561-8-S1-S85

**Published:** 2014-06-17

**Authors:** Shuang Xia, Shili Lin

**Affiliations:** 1 Department of Statistics, The Ohio State University, 1958 Neil Avenue, Columbus, OH 43210-1247, USA

## Abstract

The behavior of a gene can be dynamic; thus, if longitudinal data are available, it is important that we study the dynamic effects of genes on a trait over time. The effect of a haplotype can be expressed by time-varying coefficients. In this paper, we use the natural cubic B-spline to express these coefficients that capture the trends of the effects of haplotypes, some of which may be rare, over time; that is, at different ages. More specifically, to capture disease-associated common and rare haplotypes and environmental factors for data from unrelated individuals, we developed a method of time-varying coefficients that uses the logistic Bayesian LASSO methodology and B-spline by setting proper prior distributions. Haplotype and environmental effect coefficients are obtained by using Markov chain Monte Carlo methods. We applied the method to analyze the *MAP4 *gene on chromosome 3 and have identified several haplotypes that are associated with hypertension with varying effect sizes in the range of 55 to 85 years of age.

## Background

The Genetic Analysis Workshop 18 (GAW18) real data are family-based, consisting of cleaned single-nucleotide polymorphism (SNP) genotypes, sex, age at the time of examination, hypertension status, and smoking for up to 4 time points. Data on 157 unrelated individuals are also extracted from the families and made available for analysis. Previous studies have examined more than 50 genes for their associations with hypertension, and the number is growing [1]. Moreover, hypertension is also considered to be age-dependent; the chance of being hypertensive rises with age and the risk after midlife (eg, more than 50 years of age) is considerable [2]. Hence, in this paper, we aim to identify both genetic and environmental factors that are associated with high blood pressure, with the effects potentially varying at different ages.

This contribution concerns a haplotype-based method because haplotype procedures can be more powerful than a single SNP analysis if there are multiple causal variants interacting in *cis*-fashion, or if only SNPs in linkage disequilibrium with causal SNPs are genotyped. Rare haplotypes can result even when only common SNPs are considered. Thus, novel methods are needed not only to take the varying effects of haplotypes and environmental factors into account, but also to deal with the anomaly of rare variants. The particular environmental factor of interest is smoking, as it has been shown to be a potential risk factor for hypertension [3]; thus, we include smoking in our model in addition to sex and age.

Because the GAW18 data are collected prospectively, we first formulate a prospective likelihood. We then borrow the idea from the logistic Bayesian LASSO (LBL) approach to penalize parameters (regression coefficients) by setting up proper prior distributions [4]. We chose to follow the LBL idea because it has been shown to be capable of detecting rare associated haplotypes, albeit under a retrospective setting with fixed, rather than varying, coefficients. As such, the LBL time-varying coefficient (LBL-tvc) method developed in this paper can be considered as a generalization of the original LBL. The haplotype and environmental effect coefficients are obtained by using Markov chain Monte Carlo (MCMC) methods. By using the proper percentiles of the sampled parameters, we can also construct hypothesis tests to determine whether a haplotype or an environmental covariate is associated with the disease.

## Methods

### Data

We considered data from 153 unrelated individuals. Blood pressure measurements, age, and smoking status were available for up to 4 time points. Specifically, 40, 41, 45, and 31 individuals had measurements at 1, 2, 3, and 4 time points, respectively. Binary hypertension status is as defined in the original study: An individual is labeled as hypertensive if the systolic blood pressure is greater than 140 mm Hg, or the diastolic blood pressure is greater than 90 mm Hg, or if the individual is on antihypertensive medication at the time of examination. Individuals with incomplete genotype data for the SNPs under consideration were excluded from the analysis. However, individuals with measurements at less than 4 time points were all included because such individuals can be accommodated by our model (see below).

### Selection of 4 regions in the *MAP4 *gene

To provide a focused analysis, we only considered the *MAP4 *gene, which was associated with hypertension in previous studies. First we carried out preliminary analysis to find regions that provide at least weak evidence of association in *MAP4 *by single SNP analysis to reduce the computational burden of LBL-tvc. Specifically, we performed a logistic regression analysis for each SNP in the *MAP4 *gene and included age, sex, and smoking in the model as follows:

(1) $$\text{logit}\left(p\left(Y_{1}=1|\text{covariates}\right)\right)=\beta_{0}+\beta_{1}\text{SN}{\text{P}}_{k}+\beta_{2}\text{SMOK}{\text{E}}_{1}+\beta_{3}\text{AG}{\text{E}}_{1}+\beta_{4}\text{SEX}$$

where $Y_{1},$$\text{SMOK}{\text{E}}_{1},$$\text{AG}{\text{E}}_{1},$ and SEX are the hypertension status (1 if hypertension), smoking status, age, and sex at the first examination, respectively, and $SNP_{k}$ is the genotype at the *k*^th ^SNP. A chi-square analysis of variance (ANOVA) test is performed and the *p *value is recorded.

We choose SNPs corresponding to the 4 smallest *p *values as the anchors of our 4 regions for further analysis. Each chosen SNP and its 4 adjacent SNPs (2 on each side) form a 5-SNP-haplotype block. We used the Hapassoc software (http://cran.r-project.org/web/packages/hapassoc/index.html) to estimate haplotype frequencies, which were then used as the starting values for our MCMC analysis.

### Prospective likelihood formulation

Let $Y_{i,j}$ be the affection status and $E_{i,j}$ the smoking status of the *i*^th ^individual at the *j*^th ^examination (*i = 1,2,..., n; j = 1,2,3, j_i _≤ 4*), where *n *is the number of individuals with observed data. Further, let $Z_{i}$ be the phased (missing) haplotype pair and $S_{i}$ the sex of the *i*^th ^individual. The probability of a certain haplotype pair can be written as

(2) $$P\left(Z|\lambda \right)=a_{z}\left(\lambda \right)=P\left(Z=z_{k}/z_{k^{\prime }}|\lambda \right)=\delta_{kk^{\prime }}df_{k}+\left(2-\delta_{kk^{\prime }}\right)\left(1-d\right)f_{k}f_{k^{\prime }},$$

where $f_{1},\ldots ,f_{m}$ denote the *m *haplotype frequencies, $\delta_{kk^{\prime }}$ is the indicator function that equals to 1 if $k=k^{\prime }$, and *d *∈ (−1,1) is the inbreeding coefficient that can capture the excess or reduction of homozygosity [5]. When *d *= 0, Hardy-Weinberg equilibrium holds. The vector *λ *is the collection of all parameters. Suppose the disease statuses $Y_{i,j}$ are independent, conditional on the mean effect at time point *j*. In addition, assume that smoking status and haplotype are independent. Let Ψ be the collection of haplotype, smoking, sex, and age effect plus the parameters associated with haplotype frequencies, from which the mean effect can be constructed (see B-splines section). Then, the complete data prospective likelihood can be written as

(3) $$L_{c}\left(\Psi \right)=\left\lfloor \underset{i,j}{\prod }P\left(Y_{i,j}|E_{i,j},Z_{i},S_{i},\Psi \right)\right\rfloor \;\left\lfloor \underset{i}{\prod }P\left(E_{i,1},\Psi \right)\underset{j\geq 2}{\prod }P\left(E_{i,j}|E_{i,j-1},\Psi \right)\right\rfloor \underset{i}{\prod }P\left(Z_{i}|\Psi \right)\underset{i}{\prod }P\left(S_{i}|\Psi \right).$$

Let $\theta_{z,E}=P\left(Y=1|Z,E,S\right)/P\left(Y=0|Z,E,S\right)$. A logistic regression model leads to $\text{log}\left(\theta_{Z,E}\right)=\beta_{0}+\beta_{h}\left(t\right)X_{h}+\beta_{E}X_{E}\left(t\right)+\beta_{E,h}X_{E,h}+\beta_{s}X_{s},$ where $\beta_{h}\left(t\right)$ is the vector of haplotype effects at age t; $\beta_{E}$ is the smoking effect; $\beta_{E,h}$ is the interaction effect; and $\beta_{s}$ is the sex effect. Furthermore, $X_{h}$ is the design vector that gives the number of copies of each haplotype in Z; $X_{E}\left(t\right)$ is the smoking status at age *t *(age at examination); $X_{E,h}\left(t\right)$ is the interaction of smoking and haplotype; and $X_{s}$ is the sex.

### B-splines

We consider the natural cubic B-spline to express the haplotype effects over time. We write $\beta_{h}\left(t\right)=\overset{L+4}{\underset{l=4}{\sum }}\beta_{hl}B_{l}\left(t\right),$ where $\beta_{hl}\left(t\right)$ is the natural cubic B-spline basis function, and *L *is the number of interior knots. Because the age range is from 22 to 97, we let *L *= 2 and choose interior knots to be (40, 60) and the boundary knots to be (20, 100). We then rewrite the logistic model as

(4) $$\text{log}\left(\theta_{Z,E}\right)=\beta_{0}\overset{L+4}{\underset{l=1}{\sum }}\beta_{hl}B_{l}\left(t\right)X_{h}+\beta_{E}X_{E}\left(t\right)+\beta_{E,h}X_{E,h}\left(t\right)+\beta_{s}X_{s}=\beta_{0}+\tilde{\beta }\tilde{X}\left(t\right),$$

where $\tilde{\beta }=\left(\beta_{hl},\beta_{E},\beta_{E,h},\beta_{s}\right),$$\tilde{X}\left(t\right)=\left(B_{l}\left(t\right)X_{h},X_{E,h}\left(t\right),X_{s}\right)$. The likelihood function is now completely specified in terms of the parameter vector Ψ.

### MCMC estimation of parameters

We follow the LBL [4] methodology for estimating the parameters. A double exponential distribution with mean 0 is set to be the prior distribution for each parameter in $\tilde{\beta },$ with the intensity parameter set to be gamma, to control shrinkage. Uninformative priors are set for haplotype frequencies and inbreeding coefficient. We use MCMC methods to sample the parameters from the appropriate posterior distributions. If it is feasible to sample directly from the conditional distribution of a parameter, then we use the Gibbs sampler; otherwise, we use the Metropolis-Hastings algorithm with appropriate proposal distribution.

## Results

**Figure 1:**
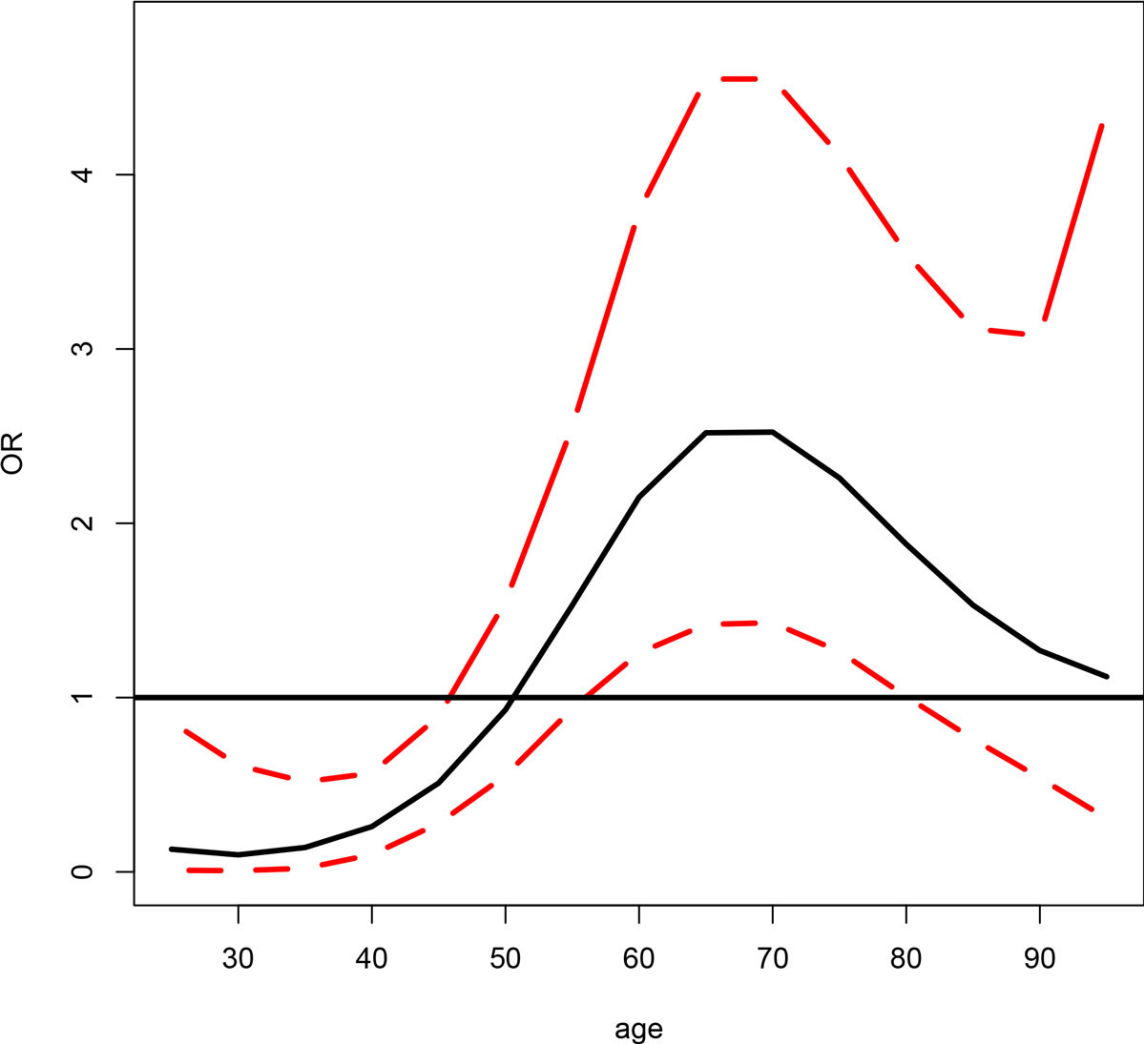
**Effect of Haplotype GGTCC (47964587 to 47985074 bp) over the specified age range**. The solid line is the odds ratio (OR) of the effects and the 2 red dotted lines are upper and lower bounds of 95% credible intervals.

LBL-tvc was applied to each of the four 5-SNP-haplotype blocks/regions in the *MAP4 *gene. For each region, at least 1 haplotype shows significant effect at the age range of 55 to 85 years (Table 1). It is interesting to see that the effect over time is consistent over all 4 regions. Specifically, the associated haplotypes do not confer risk until an individual turns 55 to 60 years of age. The risk continues to rise and reaches the maximum at an age of 70 to 75 years. To see this more clearly, we have plotted the haplotype effects over time (Figure 1) for the associated haplotype GGTCC in region 2, which spans the region from 47964587 to 47985074 base pairs (bp) on chromosome 3, covering 2 introns and 1 exon. The haplotype appears to be protective at a young age and gradually becomes a risk haplotype when an individual reaches 60 years of age. The effect is the strongest at 65 to 70 years of age, with an estimated odds ratio of 2.52, indicating a fairly strong association. The effect appears to diminish at an older age. However, note that the number of subjects in the young (25 to 40 years) and the old (90 to 95 years) categories are very small, and thus the corresponding results in these categories should be interpreted with great caution. This phenomenon also occurs in the other haplotypes. Also note that for the identified haplotype CGAGG in region 47911271 to 47915368 bp, which covers 3 exons and 3 introns, the estimated haplotype frequency is less than 0.05, considered to be a rare haplotype. The effect of this haplotype would have been overlooked had one used a pooling method by combining this haplotype with other rare haplotypes or with a common similar haplotype [4]. Although sex and smoking are also included in the model, neither effect is deemed to be significant according to our model.

**Table 1:** Significant haplotypes and their effect estimates in 4 regions of the *MAP4 *gene on chromosome 3

	47911271- 47915368(EIEIEI*)						47964587-47985074(IEI*)			47998716-48005285(I*)			48022323-48037328(I*)		
**Hap**	**CGACG†**			**TGGCG**			**GGTCC**			**TTCG**			**ATTTG**		

**Age**	**OR^‡^**	**L^‡^**	**U^‡^**	**OR**	**L**	**U**	**OR**	**L**	**U**	**OR**	**L**	**U**	**OR**	**L**	**U**

25	0.72	0.05	6.56	0.19	0.01	1.46	0.13	0.01	0.86	0.14	0.01	0.90	0.16	0.01	0.99

30	0.59	0.04	4.22	0.15	0.00	1.04	0.10	0.01	0.61	0.11	0.01	0.63	0.13	0.01	0.70

35	0.55	0.07	2.77	0.19	0.02	0.83	0.14	0.02	0.52	0.14	0.02	0.51	0.16	0.03	0.57

40	0.58	0.10	2.21	0.30	0.08	0.84	0.26	0.10	0.57	0.26	0.10	0.55	0.27	0.11	0.59

45	0.69	0.14	2.35	0.54	0.23	1.17	0.51	0.28	0.89	0.50	0.28	0.87	0.49	0.27	0.88

50	0.92	0.23	2.96	0.96	0.48	1.90	0.93	0.55	1.55	0.94	0.56	1.56	0.89	0.53	1.50

55	1.31	0.39	4.66	1.59	0.85	3.05	1.53	0.94	2.55	1.62	1.01	2.67	1.50	0.91	2.49

60	1.97	0.63	7.91	2.29	1.19	4.49	2.15	1.26	3.79	2.38	1.41	4.16	2.17	1.27	3.81

65	2.99	0.92	13.3	2.69	1.39	5.36	2.52	1.42	4.55	2.85	1.64	5.10	2.59	1.48	4.68

70	4.40	1.13	25.4	2.67	1.42	5.19	2.52	1.43	4.55	2.84	1.65	5.02	2.63	1.52	4.68

75	5.98	1.23	53.8	2.32	1.25	4.49	2.26	1.26	4.11	2.50	1.44	4.47	2.35	1.36	4.20

80	7.14	1.23	98.7	1.87	0.98	3.68	1.88	1.01	3.55	2.04	1.11	3.81	1.95	1.09	3.69

85	7.15	1.09	120	1.46	0.70	3.02	1.53	0.76	3.12	1.69	0.80	3.32	1.57	0.80	3.27

90	5.71	0.77	103	1.15	0.45	2.88	1.27	0.54	3.08	1.30	0.55	3.17	1.28	0.54	3.23

95	3.47	0.31	82.1	0.96	0.23	3.69	1.12	0.31	4.38	1.12	0.32	4.28	1.11	0.31	4.29

*In each of the 5-SNP-haplotype regions in the *MAP4 *gene, the exons (E) and introns (I) covered by the region are indicated. For example, the region from 47911271 to 47915368 covers 3 exons and 3 introns starting with an exon, indicating that the first SNP is in an exon and the last SNP is in an intron.

†This is a rare haplotype as the estimated frequency is less than 0.05.

^‡^OR (odds ratio), L (lower bound of 95% credible interval [CI]), and U (upper bound of 95% CI) are presented for ages from 25 to 95 years, in an increment of 5.

## Discussion

The longitudinal nature of the GAW18 data calls for methodology that is able to take the correlated measurements into account. Furthermore, there is a great deal of treasures that are yet to be mined from the common SNP data collected in genome-wide association studies. To this end, we have proposed LBL-tvc, a logistic regression model, to handle the correlated measurements over 4 time points. LBL-tvc considers the effects of haplotypes, which can be rare even if all the underlying SNPs are common. Application of LBL-tvc to the *MAP4 *gene yielded results that are consistent in all 4 regions of the *MAP4 *gene and appear to be useful. As one may expect, the effect of an associated haplotype would confer risk only when an individual reaches the age of 55 to 60 years, when hypertension typically strikes. The results further demonstrate the utility of the methodology for its ability to detect the effects of rare associated haplotypes.

To evaluate the performance of LBL-tvc, we carried out a preliminary simulation study with the effect mimicking that of what we see in the real data. More specifically, the simulation model considers a 5-SNP-haplotype block in which there are 5 common haplotypes and 2 rare haplotypes, with 1 of each type being associated with the hypertensive status. The strength of association across the age range of 20 to 90 years varies in a fashion similar to the pattern in the fitted real data. We also entertained an interaction effect between smoking and the common risk haplotype. Affection and smoking status are simulated at 4 time points for 250 individuals. The results, based on 100 replications, show that the type I error is well controlled, and there is overwhelming power (>90%) for detecting the common haplotype effects in the mid-age range. The power is much lower (approximately 50%), although still reasonable, for the rare haplotype effect. The power for detecting the haplotype-smoking interaction is also very high (>90%); we note, however, that the power will likely be much smaller had the interaction been with a rare haplotype. Overall, the simulation results are encouraging and to some extent validate our findings in the real data. Nevertheless, further investigation is needed to fully evaluate the properties of the method.

Because MCMC is applied for estimating the parameters, the procedure is computationally intensive. For example, analysis of each simulation replicate on a 5-SNP-haplotype block with 250 individuals and data on 4 time points as described above took about 35 minutes to complete. Therefore, our method should be primarily used for follow-up studies in interesting gene/regions. In our real data analysis, we simply use a prescreening procedure to find single SNP signals to form haplotypes with 4 neighboring SNPs. This construction of haplotype block is somewhat arbitrary. An alternative would be to select additional SNPs based on linkage disequilibrium plots.

## Competing interests

The authors declare that they have no competing interests.

## Authors' contributions

LS designed the overall study; SX wrote the software and conducted statistical analyses. Both authors wrote, read, and approved the final manuscript.
